# Assessment of Cardiovascular Disease Risk in South Asian Populations

**DOI:** 10.1155/2013/786801

**Published:** 2013-09-14

**Authors:** S. Monira Hussain, Brian Oldenburg, Yuanyuan Wang, Sophia Zoungas, Andrew M. Tonkin

**Affiliations:** Department of Epidemiology and Preventive Medicine, Monash University, The Alfred Centre, Melbourne, VIC 3004, Australia

## Abstract

Although South Asian populations have high cardiovascular disease (CVD) burden in the world, their patterns of individual CVD risk factors have not been fully studied. None of the available algorithms/scores to assess CVD risk have originated from these populations. To explore the relevance of CVD risk scores for these populations, literature search and qualitative synthesis of available evidence were performed. South Asians usually have higher levels of both “classical” and nontraditional CVD risk factors and experience these at a younger age. There are marked variations in risk profiles between South Asian populations. More than 100 risk algorithms are currently available, with varying risk factors. However, no available algorithm has included all important risk factors that underlie CVD in these populations. The future challenge is either to appropriately calibrate current risk algorithms or ideally to develop new risk algorithms that include variables that provide an accurate estimate of CVD risk.

## 1. Introduction

Cardiovascular diseases (CVD) related to atherosclerosis, including ischaemic heart disease, stroke, and peripheral vascular disease, are the leading causes of death in most of high-, low-, and middle-income countries [1]. Although low- and middle-income countries account for more than 80% of global deaths and 85% of global disability from CVD, with a few notable exceptions, knowledge and understanding of CVD risk factors are largely derived from data obtained in developed countries [2].

Atherosclerosis and its complications develop over the entire life course as a result of combined influences of lifestyle-related factors, environmental triggers, and genetic susceptibility [3]. Clinically overt CVD is usually preceded by the presence of one or more risk factors and subclinical atherosclerosis [3], and the relationship between risk factors and CVD outcomes represents a continuum. As the individual risk varies greatly, methods that estimate the risk of future CVD events have been developed and applied. Such algorithms recognise the continuous relationship between levels of biomedical and lifestyle variables and future CVD events, weigh risk factors according to their importance, and allow for potential synergistic effects of abnormalities in individual risk factors. Such scores not only stratify individual risk and identify those most likely to benefit from an intervention, but also inform the most cost-effective allocation of preventive therapies.

A risk scoring method should include appropriate risk factors, be accurate, and be ideally validated in the population in which it is applied. Importantly, it might be also favourably influencing clinical behaviour. We anticipated that risk estimation methods widely used in low- and middle-income countries albeit sometimes calibrated using local risk factor prevalence and disease incidence data might have been developed from studies of populations based in high-income countries [2]. However to our knowledge, such calibration had not been undertaken in South Asian populations, which include Afghanistan, Bangladesh, Bhutan, India, Maldives, Nepal, Pakistan, and Sri Lanka, which are estimated to contain more than 1.64 billion people, in excess of 24% of the world's current population [4].

This paper examines the implications of CVD risk algorithms because of the potential limitations in current CVD risk assessment approaches for prevention and control of atherosclerotic CVD in South Asian populations.

## 2. Methods

MEDLINE, Google scholar, and Embase were searched using the MeSH terms and free texts for the key words, such as “cardiovascular”, “coronary”, “heart disease”, “risk equation”, “risk algorithm”, “risk predictions”, “risk scores”, “risk engines”, “risk assessments”, “risk factors”, and “South Asians”, from 1970 until July 2012. To identify other studies which also addressed the above questions, relevant book chapters and other manuscripts were also examined after citation tracking. 

A study was eligible for review if it fulfilled any of the following criteria: (1) if it compared CVD risk profiles between South Asians and Caucasians or between South Asians in their home country and South Asians living abroad; (2) if it assessed CVD risk factors that are prevalent among South Asians; (3) if it formed at least part of the evidence base for the development of a CVD risk score/equation; (4) if it examined the use of CVD risk tool(s) in South Asians. 

**Figure 1:**
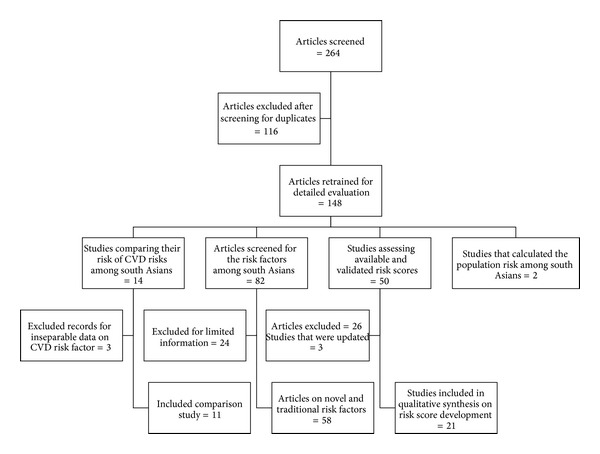
Flow diagram for the included articles.

Details concerning inclusion and exclusion of studies are outlined in Figure 1. Two reviewers (SMH and YW) extracted information concerning study demographics, the predictive variables examined, and clinical end-points for each study.

## 3. Results

### 3.1. CVD Risk Profiles in South Asians Compared with Those in Caucasians and South Asians Living in Other Countries

The diversity and differences in CVD risk factors in native South Asians compared to South Asians who have migrated abroad and between the South Asians and other ethnic groups (Caucasians and Chinese) are shown in Table 1. There were marked differences in the distribution of CVD risk factors [5–7]. It was concluded that the wide array of differences in CVD risk profile makes combining data from different misleading South Asian countries [6, 7]. Even within countries such as India, marked variations in risk profiles due to difference in culture, lifestyle, and religion have been reported. Nevertheless, all of these studies have concluded that despite their lower body mass index (BMI), body weight, and serum cholesterol, South Asians have greater evidence of other features of dyslipidaemia including lipoprotein(a) and other CVD risk factors than Caucasians [5–10]. Additionally, South Asians had more prominent abnormalities in fibrinogen, plasminogen activator inhibitor 1, homocysteine, C-reactive protein, insulin sensitivity, and psychosocial factors (stress, depression) [6–9]. 

### 3.2. Assessment of CVD Risk Factors in South Asians in Their Native Countries

The widely discussed CVD risk factors in South Asians are listed in Table 2. The INTERHEART study and other recent researches in Singapore, the United Kingdom, and Canada, which examined large populations from South Asian countries [7, 8, 10, 11], reported that South Asians tended to experience both the “classical” and nontraditional CVD risk factors in a higher level and at a younger age than the other populations [7, 8, 11, 12]. For example, South Asians were more likely to have more severe abnormalities of harmful factors—including elevated apo B100/apo A-I ratio [7, 11, 13], lipoprotein(a) [8, 11, 14], depression, and stress at work or home [7, 13], and had lower rates of protective factors—physical activity [7, 13], regular fruits, and vegetable intake [7, 13]. Concurrently, potentially widespread environmental pollution, such as that from arsenic contamination and indoor air pollution, further complicates the situation. Arsenic exposure which is associated with vascular inflammation and endothelial dysfunction exerted by atherosclerotic plaque, oxidative stress, and lipid peroxidation [15] is a huge problem for an estimated 40–60 million people in the Ganges delta of South Asia [16] as they consume arsenic contaminated groundwater [16] and food from plant origin [17]. Most of the rural houses in Bangladesh [18] and 75% of households of India [19] use solid fuels for cooking which are the major sources of indoor air pollution and pose serious health risks including CVD and low birth weight [18, 19]. 

With 31% of all infants born with low birth weight, South Asians had the highest incidence of this problem [20] which has been consistently associated with increased risk of insulin resistance and atherosclerotic CVD later in life [21, 22]. It has been postulated that low birth weight contributes to the early development of the CVD risk factors, including blood pressure and plasma glucose concentrations, insulin resistance, abnormal fibrinogen/factor VII, and apolipoprotein levels [23] that are common among South Asians. 

### 3.3. Currently Available and Validated Risk Algorithms

Table 3 summarizes details concerning the most widely used risk scores, the populations from which they were derived, the risk factors included, and the different CVD outcomes which were examined. A person's estimated probability of developing a major CVD event(s) during the following 5-or 10-year period is known as absolute or short-term risk. Absolute risk calculators are developed and validated in longitudinal cohort studies which estimate the effect of multiple CVD risk factors on CVD events [24]. However, if any CVD risk factor was left untreated for a long time, it can produce high risk and cumulative damage [25]. Not only for this reason but also because age is the major determinant of short-term absolute risk a lifetime risk, scoring method has been developed and has been recommended for younger patients to offer additional risk burden.

The relationship between independent major risk factors and coronary heart disease (CHD) risk was first described over 50 years ago using data from the Framingham Heart study. Since then, over 100 different CVD risk scores have been developed, mainly based on findings on Caucasians in developed countries. Among the most commonly used risk scores, at least five have been derived from the Framingham study [26–28]. Other studies from which risk scores have been derived include those of the Munster group (PROCAM) [29], ARIC [30], QRISK (electronic medical database) [31, 32], Reynolds [33, 34], the Scottish Heart Health Extended Cohort [35], Strong Heart Study [36], USA-PRC (People's Republic of China Collaborative Study of Cardiovascular Epidemiology) [37], the SCORE project [38], Personal heart [39], NHEFS [2], and the UKPDS [40]. None of the above risk scores have been derived from epidemiologic studies undertaken in developing countries and/or a South Asian population living in South Asia. Although the IHMRS (obtained from INTERHEART data) has been validated in South Asians, it has the limitation of being derived from a case-control study which might not be considered to be as robust in predicting future events as equations derived from prospective data [41].

The numbers of risk factors that incorporated into the risk scores have varied widely ranging from 4 to 14. All algorithms have included age, gender, blood pressure, and smoking status, and also they almost included lipid parameter(s) and diabetes status. Of note, lipid parameters were not included in the nonlaboratory model of the 2008 Framingham risk score [28], the PROCAM 2007 (stroke) risk score [29] or the NHEFS risk score [2]. The CVD outcome(s) predicted by the risk scores also differs markedly, as again shown in Table 3.

### 3.4. Calculation of CVD Risk with Currently Available Risk Tools

There is no specific risk score that has been derived from South Asian populations; nor to our knowledge has any attempt been made to calibrate either short-term or lifetime risk stratification algorithms in these populations. 

Some of the currently available risk scores have been applied to calculate CVD risk in South Asians. For example, when the ATP III (Adult Treatment Panel) risk score (based on the Framingham study) was applied to a cohort of healthy Indian industrial workers of 40.8 ± 10.9 years of age on average (range 20–69 years), it was estimated that one in two men and three in four women had a low short-term risk for CVD [42]. Based on this aggregate of risk factor burden, these results highlight a key limitation—an overestimation of CVD risk [42]. As with almost all of the combinations of risk factors at their extreme values, the ATP III risk score predicts 10-year CVD risks below 10% for nonsmoking men <45 years of age and all women <65 years of age [43]. In another Indian cohort, three risk scores were applied and predicted different levels of CVD risk for the individuals in the same population [12]. The Framingham model predicted only 5.3% of the study subjects (males aged 37.2 ± 14 years and females aged 43.2 ± 13.7 years) at high risk; an apparent underestimation [12] has given that an adjusted Framingham score calculates 30-year risks to be 7.9% for women and 18.0% for men in the population from which it originated [44]. That Framingham algorithm has been observed to perform poorly across socioeconomic groups, and underestimated risk in socioeconomically deprived groups [45] may be an explanation for this underestimation. The Joint British Cardiac Society (BCS)/British Hypertension Society (BHS)/British Hyperlipidemia Association (BHA) risk score also appeared to underestimate the CVD risk [12]. However, the risk underestimation by the Framingham algorithm and the British Cardiac Society algorithm was disproportionate (5.3% by Framingham, 3.7% and 4.4% by the joint British Societies scores, resp.) [12]. 

## 4. Discussion

This review has shown that the distribution of CVD risk factors varies markedly in South Asians. Along with the conventional CVD risk factors, some influential nontraditional risk factors which are more common among these populations may be relevant in explaining why South Asians have CVD risk in a higher level and at a younger age. Studies have found that the predictability of CVD risk scores, which were developed mainly for Caucasians, varies considerably mainly by underestimating lifetime risk among South Asians. To overcome this underestimation, the recent UK lipid-lowering guidelines suggest that absolute risk estimated with an earlier version of the Framingham CVD score should be multiplied by 1.4 for men of South Asian origin [46]. However, this multiplication has the possibility of incorrect adjustments as South Asians possess a number of heterogeneous CVD risk factors as already discussed. 

Although the classical CVD risk factors such as age, smoking, dyslipidaemia, elevated blood pressure, and abnormal glucose metabolism apply to South Asians as they do to Caucasians, Chinese and African populations, the British Cardiac Society among others has warned against assuming that risk scoring methods developed in non-South Asian populations can be directly applied to South Asians [46]. This is partly because data regarding incident CVD mortality and morbidity and risk factor prevalence are inadequate for most South Asian populations, therefore not allowing calibration of a CVD risk score derived from another population. Moreover, there was no local longitudinal cohort to supply robust information on the relation between CVD risk factors and outcomes. As a result, the extent to which the “classical” risk factors actually explain the rates of CVD among South Asians is still largely unanswered.

### 4.1. Chronological Age and CVD Risk

Although chronological age partly influences the formation of atherosclerotic plaque, it does not reflect true cardiovascular age [47]. As a result, assigning the same CVD risks to all individuals of the same chronologic age will be problematic because of the great variation in plaque burden at that point in a lifetime [47]. Yet, age remains one of the key variables in CVD risk scores. The sensitivity of the Framingham risk score and SCORE was lower in the younger age group (40–45 years) and older age group (70–85 years) compared with that in the middle age group (55–64 years) [48] even when risk factor levels were unfavourable among the younger persons. For example, the Framingham score nearly reached 10% risk in people aged 18–29 years and calculated risk <12% in people aged 30–39 years despite the fact that the risk factor burden was remarkably high in these young individuals. In the INTERHEART study, hypertension, smoking, dyslipidaemia, and diabetes in women <60 years of age were more strongly associated with myocardial infarction than the presence of the same risk factors in women >60 years of age [13]. A concerning fact for South Asians is that CVD risk for this group should be estimated at least 10 years earlier than in Caucasians and Chinese as these people harbinger CVD risk factors at a younger age [7, 13]. Additionally, there may be variations in the extent of atherosclerosis due to not only “conventional” but also novel risk factors which can partly explain their higher rates of CVD [8].

### 4.2. Migration and Changes in CVD Risk Factors

Migration is associated with changes in lifestyle, psychosocial interaction, dietary habit, climate, culture, and living conditions, which ultimately may influence the CVD risk profile. However, the change in CVD risk depends on the country which migration has taken place from and the country to which people have migrated. Most studies have reported that migration has a deleterious effect on CVD risk factors. For example, migrants from the Indian subcontinent who settled in the USA, UK, or other European countries have been reported to have increased obesity, apolipoprotein B levels [7, 11, 49], fasting plasma insulin [11, 49], C-reactive protein [49], serum triglyceride [50], and decreased *β* cell functions [11, 49] compared to the natives of those countries [11, 49–51]. In addition, migration could be associated with significant psychosocial stress that may impact on CVD, though such evidence is sparse [52]. All these factors make it questionable whether the risk score derived from the Strong Heart study (undertaken in American Indians) or ARIC and UKPDS algorithms in which ethnicity is considered as one of the risk factors can be transferred directly for use in the South Asian populations. 

### 4.3. Central Obesity and Insulin Resistance in South Asians

Several explanations including the role of body composition have been proposed to explain the paradox between the high prevalence of diabetes and atherosclerotic CVD despite a lower mean BMI and waist circumference among South Asians. Studies have shown that for a given BMI or waist circumference South Asian males had approximately 6% higher total body fat than Caucasian men [52]. Additionally, South Asians had larger abdominal subcutaneous adipocytes than Caucasians, a phenomenon associated with insulin resistance independent of waist circumference. Several longitudinal studies have demonstrated that individuals who are insulin resistant develop abnormal CVD risk factors long before the development of overt diabetes [41, 52]. These abnormal CVD risk factors include low adiponectin and altered adipokines levels, lipid abnormalities [41], glucose intolerance, elevated blood pressure [41, 52], chronic inflammation [52], and an increased procoagulant state [41] that ultimately lead to microvascular and macrovascular complications [41, 52, 53]. Risk assessment is a useful tool in CVD prevention as it promotes the most effective and cost-effective use of preventive strategies and highlights the importance of lifestyle modification. It may also be used as a motivational tool according to the manner in which it is presented. South Asian populations are well known to suffer an excessive and premature burden of CVD. But, estimating their CVD risk accurately is difficult as the popular risk algorithms either underestimate [12] or overestimate the CVD risk [42], and different algorithms often predict CVD risk differently in the same individual [12]. 

### 4.4. Strengths and Limitations

This review is a qualitative synthesis of published studies which used different epidemiological designs with varying sample sizes and did not adhere only to the hierarchy of evidence. However, the literature search was systematic, and the qualitative synthesis that was made was robust. Moreover, this study provides complete information on prevailing CVD risk factors and the vulnerability of the South Asians to CVD, the CVD risk factors difference between South Asians and Caucasians and others, and the risk factors included in the currently available risk scores and their prediction of CVD risk among these populations.

## 5. Conclusion

For South Asians, the risk scoring method should include a range of variables that provide an accurate estimate of risk within complex population structures with differences in sociodemographic groups, comorbidities, culture, environmental features, linguistics, and population dynamics. Final challenges include how to assess risk in younger individuals and how to better individualise risk rather than to stratify risk within subgroups of the adult population. This is now being assessed by the extent to which new risk factors/markers appropriately reclassify risk between those who do and do not develop CVD [54]. 

## Figures and Tables

**Table 1:** Variations of risk factors in South Asians in their home countries or migrant South Asians compared with the other populations.

Author & year	Study design	Study population	Risk factors estimated	Conclusion
Bhatnagar et al. [11] 1995	Cross-sectional	Total 364 British Punjab 247, Indian Punjab 117	BMI, BP, TC, TG, HDL, apo B, Lp(a), insulin sensitivity, *β* cell function, blood glucose	(i) Indian migrants in the UK have a less favourable coronary risk profile (ii) Indians have higher serum Lp(a) which is not affected by migration (iii) Deterioration of insulin resistance and *β* cell function is associated with migration

Cappuccio et al. [5] 1997	Cross-sectional	Total 1578 British white 524, British African 549, British South Asian 505	BP, diabetes, BMI, serum cholesterol, smoking	(i) South Asians have a higher prevalence of hypertension and diabetes than Caucasians (ii) One in five people of South Asian origin had diabetes (iii) CHD is the first cause of death among South Asians (iv) There are marked differences in the distribution of CVD risk factors among Caucasians, South Asians and Africans

Bhopal et al. [6] 1999	Cross-sectional	Total 1508 684 South Asians (British Indian 259, British Pakistani 305, British Bangladeshi 120), British Europeans 825	TC, HDL, LDL, TG, Lp(a) lipoprotein, fibrinogen, insulin, and glucose, Height, weight, waist and hip, BP, ECG, smoking, diet, alcohol consumption, exercise	(i) South Asians have more CHD than Europeans despite apparently lower levels of risk factors (ii) Indians, Pakistanis and Bangladeshis differ in a wide range of coronary risk factors (iii) Among South Asians, Indians were least and Bangladeshis were most disadvantaged in a range of coronary risk factors (iv) Combining the data on CVD risk factors from Indians, Pakistanis and Bangladeshis is misleading

Anand et al. [8] 2000	Cross-sectional	Total 985 South Asians 342, European 326, Chinese 317	BP, BMI, waist : hip ratio, LVH, TC, LDL, HDL, TG, blood glucose, Lp(a), homocysteine, PAI-1, fibrinogen, smoking, subclinical atherosclerosis	(i) Asians had more plasma lipid and glucose abnormalities (ii) South Asians had the highest prevalence of CVD and highest DBP (iii) South Asians had greater abnormalities in novel risk factors: fibrinogen, PAI-1, Lp(a) and homocysteine

Chambers et al. [9] 2001	Cross-sectional	Total 1025 males Indian Asians 518, European whites 507	Age, CRP, BMI, Waist : hip ratio, BP, smoking, physical activity, blood glucose, TC, HDL, TG, insulin resistance	(i) CRP levels are elevated in Indian Asians and are associated with an increase in population CHD risk among Indian Asian (ii) Diabetes, insulin resistance and related metabolic abnormalities are more common among Indian Asians (iii) CRP levels were more closely associated with increased central adiposity and markers of insulin resistance

Mahajan and Bermingham [14] 2004	Cross-sectional	Total 250 Australian Indians 125, Indian Indians 125	Age, BMI, Waist, Waist : hip ratio, Smoking, alcohol consumption, food habit, exercise, TC, HDL, TG, LDL, Apo A1, Apo B, Lp(a), Insulin	(i) Indians migrated to Australia have a more favourable CHD risk factor profile than the Indians remaining in India (ii) It is unlikely that changes due to migration have a strong genetic bias

Patel et al. [49] 2006	Cross-sectional	Total 536 British Gujarati 242, Indian Gujarati 294	Age, education, smoking, alcohol intake, height, BMI, Waist : hip ratio, ECG, BP, blood glucose, physical activity, energy intake, folate, vit B12, energy from fat & carbohydrate, TC, TG, NEFA, HDL, LDL, Apo B, Apo AI, Apo B : AI ratio, Lp(a), CRP, Homocystine, Serum folate & vit B12, Plasma insulin, HOMA B, HOMA S	(i) Migration adversely influences some of the CVD risk factors, like—apo B, plasma insulin, CRP (ii) Migration makes British Gujaratis more prone to CVD than Indian Gujaratis

Joshi et al. [7] 2007	Case-control	Total 29,972 (Case + control) India 470 + 940, Pakistan 637 + 655, Sri Lanka 153 + 132, Bangladesh 228 + 238, Nepal 244 + 239 Other countries 13420 + 12616	Age, SES, tobacco use, alcohol, physical activity, dietary patterns, personal or family history of CVD, hypertension, stress and depression, Height, weight, waist : hip ratio, apo B100 and apoA-I, ApoB100 : ApoA-I ratio, diabetes	(i) Participants of South Asian countries experience fatal CHD at younger ages (ii) Variations in the mean age of presentation of cases were observed between countries within South Asia (iii) Bangladeshis had the highest prevalence for the most of the CVD risk factors among controls (iv) Indians have the higher prevalence of diabetes

Tennakoon et al. [50] 2010	Cross-sectional	Total 1378 Norwegian Sri Lankans 1145, Sri Lankans in Sri Lanka 233	Age, education, smoking, TC, HDL, TC : HDL ratio, TG, Height, BMI, Waist circumference, BP	(i) Norwegian Sri Lankans have favourable lipid profiles and blood pressure levels despite being more obese compared with people living in Sri Lanka (ii) Educated persons in Sri Lanka are in more risk of having higher triglyceride, obesity, and blood pressure compared to the uneducated people

Chiu et al. [10] 2010	Cross-sectional	Total 163,797 Canadian White 154653, Canadian South Asians 3364, Canadian Chinese Total 3038, Canadian black people 2742	Age, education, sex, current smoking, obesity, Diabetes, BP, stress, consumption of fruits and vegetables, physical activity, non-regular alcohol consumption	(i) There are differences in the prevalence of CVD risk factors across ethnic groups (ii) Canadian South Asians are less obese than whites, but they suffers more from diabetes and hypertension (iii) The protective factors like fruit and vegetable consumption, exercising habit, are less common in Canadian South Asians

Zahid et al. [51] 2011	Cross-sectional	Total 2000 Norwegian Pakistani 770, Pakistanis living in Pakistan 1230	Age, height, weight, BMI, Waist circumference, Hip circumference, Waist : hip ratio, smoking, TC, HDL, BP	(i) Obesity and CVD risk factors are widely prevalent in both Norwegian Pakistani and Pakistanis living in Pakistan (ii) Migration makes Norwegian Pakistani more likely to have CVD risk factors (obesity and dyslipidemia) compared to Pakistanis at their homeland

BMI: Body mass index, BP: Blood pressure, TC: Total cholesterol, HDL: High density lipoprotein, LDL: Low density lipoprotein, TG: Triglyceride, apo: Apolipoprotein, Lp: lipoprotein, ECG: Echo cardiogram, LVH: Left ventricular hypertrophy, PAI: Plasminogen activator inhibitor, CRP: C reactive protein, vit: Vitamin, NEFA: Nonesterified fatty acids, HOMA: homeostatic model assessment.

**Table 2:** Widely distributed cardiovascular disease risk factors among South Asians.

Traditional risk factors	Nontraditional risk factors
*Nonmodifiable* (i) Age (ii) Sex (iii) Familial *Modifiable* (i) High blood pressure (ii) Diabetes (iii) Dyslipidaemia (iv) Abdominal obesity (v) Smoking	*Nonmodifiable* (i) Low birth weight [21, 22, 55] (ii) Abnormal foetal growth [21, 22] *Modifiable* (i) Insulin resistance [6, 9, 11, 14, 49, 56] (ii) High apo B100/apo A-I ratio [7, 11, 13, 14] (iii) Low adiponectin [8] (iv) Lipoprotein (a) [6, 8, 11, 14] (v) Homocysteine [8, 49] (vi) Apolipoprotein B [7, 11, 14, 49] (vii) Visceral and ectopic fat [13] (viii) Plasminogen activator inhibitor-1 [8] (ix) Fibrinogen [8] (x) Endothelial dysfunction (xi) Low physical activity [6, 7, 10, 14, 49] (xii) Dietary habit [7, 10, 13] (xiii) Alcohol consumption [6, 7, 10, 13] (xiv) Psychosocial factors [7, 10, 13]

**Table 3:** Key characteristics of important cardiovascular risk algorithms.

Risk scores	Country	Population	Risk factors	Outcomes
Framingham [26] 1976	USA	Population cohort	Age, sex, smoking, SBP, total cholesterol, random blood sugar and urinary blood glucose, ECG-LVH	Deaths from CHD and other fatal CVD, MI, ischaemic stroke, haemorrhagic stroke, angina pectoris, PAD, hypertensive CCF

Framingham [27] 1991	USA	Population cohort (original + offspring)	Age, sex, smoking, SBP, DBP, TC, HDL, RBS, (offspring FBS) ECG-LVH	Death from CHD, MI, angina pectoris

Framingham [57] 1998	USA	Population cohort (original + offspring)	Age, sex, smoking, SBP, DBP, TC or LDL, RBS, (offspring FBS)	Death from CHD, MI, angina pectoris

Framingham [28] 2008	USA	Population cohort (original + offspring)	Lab based-Age, sex, smoking, SBP, TC, HDL, FBS, antihypertensive	Deaths from CHD and other fatal CVD, MI, ischaemic stroke, haemorrhagic stroke, TIA, angina pectoris, PAD, hypertensive CCF
			Non-lab-Age, sex, smoking, SBP, FBS, antihypertensive, BMI	

UKPDS [40] 2001	UK diabetes prevention trial	Population cohort	Age at diagnosis of diabetes, smoking, SBP, TC/HDL, HbA_1c_, ethnicity	Hard CHD

PROCAM [29] 2007	Germany	Occupational cohort	CHD-Age, sex, smoking, SBP, LDL, HDL, TG, FBS, family history	Deaths from CHD and MI
			Stroke-Age, sex, smoking, SBP, FBS	Ischemic stroke, TIA

SCORE [38] 2003	Europe	Pooled dataset of cohort studies	Age, sex, smoking, BP, TC or TC : HDL	Deaths from CHD and other fatal CVD, fatal MI, fatal ischemic score, fatal PAD, fatal hypertensive CCF

ARIC [30] 2003	USA	Population cohort	Age, sex, smoking, SBP, TC, HDL, FBS, antihypertensive, ethnicity	Death form CHD, MI, revascularisation intervention

Dubbo equation [58] 2003	Australia	Second Australian national blood pressure study	Age, sex, smoking, SBP, antihypertensive medication, TC, HDL, diabetes	CVD

Progetto coure [59] 2004	Italy	Pooled dataset of cohort studies	Age, sex, smoking, SBP, TC, HDL, FBS, antihypertensive	Deaths from CHD and other fatal CVD, MI, ischemic stroke, haemorrhagic stroke, revascularisation intervention

DECODE [60] 2005	Sweden	National diabetes registry	Age, state of glucose tolerance, FBS, smoking, SBP, TC, HDL, BMI, HR	Fatal CVD and first incident CVD

Riskard [61] 2005	Italy	Population cohort	Age, sex, smoking, mean BP, HDL-C, non-HDL-C, diabetes, BMI, heart rate	CVD, CHD and stroke

Zhang et al. [62] 2005	China	Occupational cohort	CHD-Age, smoking, SBP, TC, BMI	CHD
			Ischemic stroke-age, smoking, SBP, TC	Ischaemic stroke
			Haemorrhagic stroke-age, SBP, DBP, TC	Haemorrhagic stroke

Strong Heart Study [36] 2006	USA	Population cohort-American Indian	Age, sex, smoking, SBP, TC or LDL, HDL, FBS, antihypertensive, albuminuria	Death from CHD, MI, angina pectoris, revascularisation interventions

USA-PRC [37] 2006	China	Population cohort	Age, sex, smoking, SBP, TC, FBS, BMI	Deaths from CHD and other fatal CVD, MI, ischemic stroke

NIPPON data80 [63] 2006	Japan	Population cohort	Age, sex, smoking, SBP, TC, blood glucose	Fatal CHD, fatal stroke, and fatal CVD

ASSIGN [35] 2007	UK	Population cohort	Age, sex, smoking, SBP, TC, HDL, diabetes, family history, socioeconomic status	Deaths from CHD and other fatal CVD, MI, revascularisation intervention, hypertensive CCF and; admitted for MI, ischemic stroke, haemorrhagic stroke, TIA or angina pectoris

Reynolds women [33] 2007	USA	Women's health Study trial subjects	Age, smoking, self-reported BP, TC, HDL, HbA_1c_ (if diabetic), family history, hs-CRP	Deaths from CHD and other fatal CVD, MI, ischaemic stroke, revascularisation intervention

Reynolds men [34] 2008	USA	Physician health study trial subjects	Age, smoking, Self-reported BP, TC, HDL, diabetics excluded in baseline, family history, hs-CRP	Deaths from CHD and other fatal CVD, MI, stroke, revascularisation interventions

Personal heart [39] men 2007	USA	Population cohort	Men age, smoking, previous diagnosis of HTN, previous diagnosis of hypercholesterolemia, previous diagnosis of diabetes, family history, physical activity Women age, smoking, previous diagnosis of hypertension, previous diagnosis of hypercholesterolemia, previous diagnosis of diabetes, BMI	Death from CHD, MI, revascularisation intervention

QRISK [31] 2007	UK	Electronic medical database	Age, sex, smoking, SBP, TC : HDL, diabetics excluded in baseline, antihypertensives, BMI, family history, townsend	Deaths from CHD and other fatal CVD, MI, stroke, TIA, angina pectoris

QRISK2 [32] 2008	UK	Electronic medical database	Age, sex, smoking, SBP, TC : HDL, FBS, antihypertensive, BMI, ethnicity, family history, townsend, rheumatoid arthritis, chronic renal disease, atrial fibrillation	Deaths from CHD and other fatal CVD, MI, ischaemic stroke, TIA, angina pectoris

NHEFS [2] 2008	USA	Population cohort	Age, sex, smoking, SBP, previous diagnosis of diabetes, BMI	Deaths from CHD and other fatal CVD, MI, stroke, revascularisation interventions, Hypertensive CCF

IHMRS [41] 2011	52 countries	Cases of MI, age (±5 years), and sex-matched controls	Age, apolipoprotein B : A1 ratio, smoking, second-hand smoke, diabetes, high blood pressure	Cases of acute MI

SBP: systolic blood pressure, TC: Total cholesterol, HDL: High density lipoproetein, LDL: low density lipoprotein, TG: triglyceride FBS: Fasting blood sugar, HR: Heart rate MI: myocardial infarct, TIA: transient ischemic attack, PAD: peripheral artery disease, CCF: congestive cardiac failure.
